# Magnetoelectricity of CoFe_2_O_4_ and tetragonal phase BiFeO_3_ nanocomposites prepared by pulsed laser deposition

**DOI:** 10.1038/s41598-017-18788-8

**Published:** 2018-01-10

**Authors:** Min Gao, Ravindranath Viswan, Xiao Tang, Chung Ming Leung, Jiefang Li, D. Viehland

**Affiliations:** Department of Materials Science and Engineering, Virginia Tech, Blacksburg, VA 24061 USA

## Abstract

The coupling between the tetragonal phase (T-phase) of BiFeO_3_ (BFO) and CoFe_2_O_4_ (CFO) in magnetoelectric heterostructures has been studied. Bilayers of CFO and BFO were deposited on (001) LaAlO_3_ single crystal substrates by pulsed laser deposition. After 30 min of annealing, the CFO top layer exhibited a T-phase-like structure, developing a platform-like morphology with BFO. Magnetic hysteresis loops exhibited a strong thickness effect of the CFO layer on the coercive field, in particular along the out-of-plane direction. Magnetic force microscopy images revealed that the T-phase CFO platform contained multiple magnetic domains, which could be tuned by applying a tip bias. A combination of shape, strain, and exchange coupling effects are used to explain the observations.

## Introduction

Magnetoelectricity (ME) is a coupling between magnetic and polar order parameters^1^. It makes possible the manipulation of the magnetic spins by an applied electric field, offering potential for sensors and spintronic devices^2–4^. Such ME coupling has been reported in both single-phase and composite materials, which has attracted significant interest around the world^2,5^. Normally, single-phase ME materials are considered as multiferroics with two coexisting orders. Strain-mediated ME composites made of magnetostrictive and piezoelectric materials have a much stronger ME coupling, relative to single-phase systems.

The spinel CoFe_2_O_4_ (CFO) is a typical ferrimagnetic oxide that has a high saturation magnetization, high Curie temperature, and large magnetocrystalline anisotropy^6–8^. Combined with its large magnetostriction coefficient (λ_100_: −250 to −590 × 10^−6^), CFO is a good choice for the magnetostrictive phase in ME composites^8,9^. Alternatively, BiFeO_3_ (BFO) is the only known single phase room temperature multiferroic material. It has coexisting antiferromagnetic (Neel temperature T_N_ ~ 643 K) and ferroelectric (Curie temperature T_C_ ~ 1103 K) orders^10,11^, leading to a weak linear (intrinsic) ME coupling *via* the Dzyaloshinskii–Moriya interaction^12^. When used in a composite material, the polarization of BFO can couple to the magnetostriction of CFO, resulting in large quadratic (extrinsic) ME coupling *via* strain^13^. Since BFO is antiferromagnetic, at its interface with other ferromagnetic materials, an exchange coupling exists^14^. The coupling between BFO and other ferromagnetic metals or alloys, such as Co^15,16^ and CoFe^17^, has been considered. However, such oxide/metal heterostructures are not ideal due to incompatibilities between dissimilar material types^18^. To overcome this limitation, all-oxide systems have been proposed, such as Fe_3_O_4_/BFO^18^ and La_0.67_Sr_0.33_MnO_3_/BFO^19^. Accordingly, an exchange coupling can be expected in CFO/BFO nanocomposites at their ferrimagnetic/antiferromagnetic interfaces^20^.

Nanocomposites of CFO/BFO self-assemble into a vertically integrated nanopillar structure that results from an immiscibility and lattice mismatch between CFO and BFO phases^7,13^. All prior investigations of self-assembled nanocomposites have focused on the rhombohedral phase (R-phase) of BFO with CFO^7,13,20,21^. A tetragonal phase (T-phase) of BFO is also known to exist when deposited on LaAlO_3_ (LAO) substrates, which has a very high c/a ratio^22^. However, CFO/T-phase BFO nanocomposites have not yet been reported. The T-phase of BFO exhibits unique property characteristics, relative to the R-phase. For example, the antiferromagnetic order in the T-phase changes from G-type (R-phase) to C-type, while the ferroelectric polarization direction rotates from [111] (R-phase) to [001]^21–24^. Here, CFO/T-phase BFO nanocomposites were fabricated on (001) LAO substrates by pulsed laser deposition (PLD), and its ME properties were compared with that of CFO/R-phase BFO ones on (001) SrTiO_3_ (STO) substrates.

## Results and Discussion

**Figure 1:**
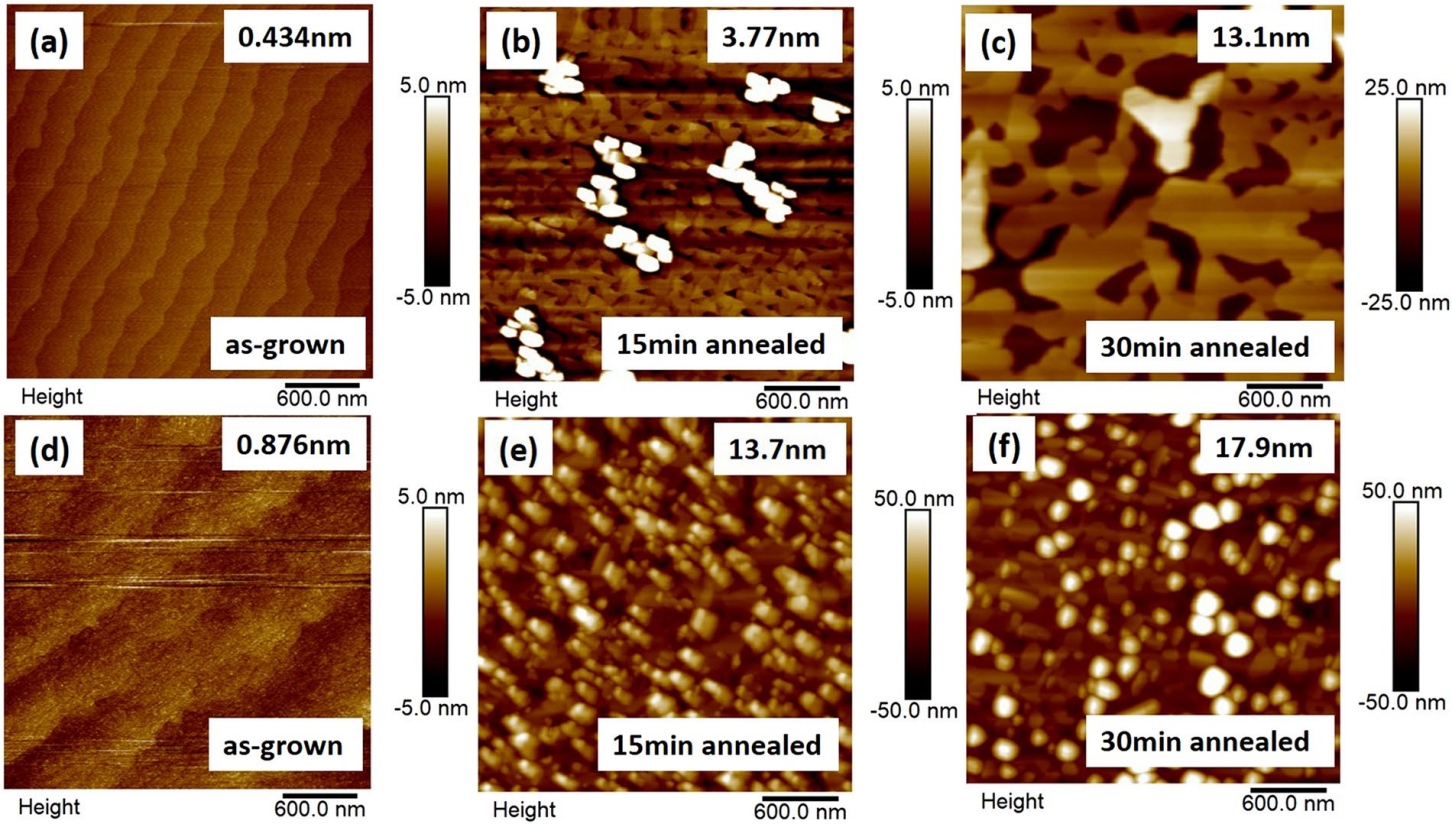
AFM height images of (**a**–**c**) CFO(7 nm)/BFO(28 nm)/(001)LAO, and (**d**–**f**) CFO(7 nm)/BFO(28 nm)/(001)STO. (**a**,**d**) The as-grown samples, (**b**,**e**) the 15 min annealed samples, (**c**,**f**) the 30 min annealed samples. The surface roughness is listed as insets.

First, the effect of annealing on the CFO/BFO nanocomposite was investigated. Figure 1 shows atomic force microscope (AFM) height images of samples having different annealing conditons. The as-grown CFO/BFO nanocomposites on both LAO and STO substrates exhibited smooth surfaces with a roughness of less than 1 nm. The wave-like topography indicates a layer-by-layer growth of the thin films. After 15 min of annealing, the smooth surfaces began to develop into specific topographies that had an obvious increase in roughness. After 30 min of annealing, the surface topography was completely transformed into different morphological patterns. Platform-like structures were observed for CFO/BFO nanocomposites on (001) LAO, and a typical nanopillar structure was found for those on STO^21^. The profile images in Figure S1 indicate that there are three distinguishable platform heights for the CFO/BFO nanocomposites on LAO substrates, where each platform has a relatively flat surface with a local roughness of less than 1 nm.

**Figure 2:**
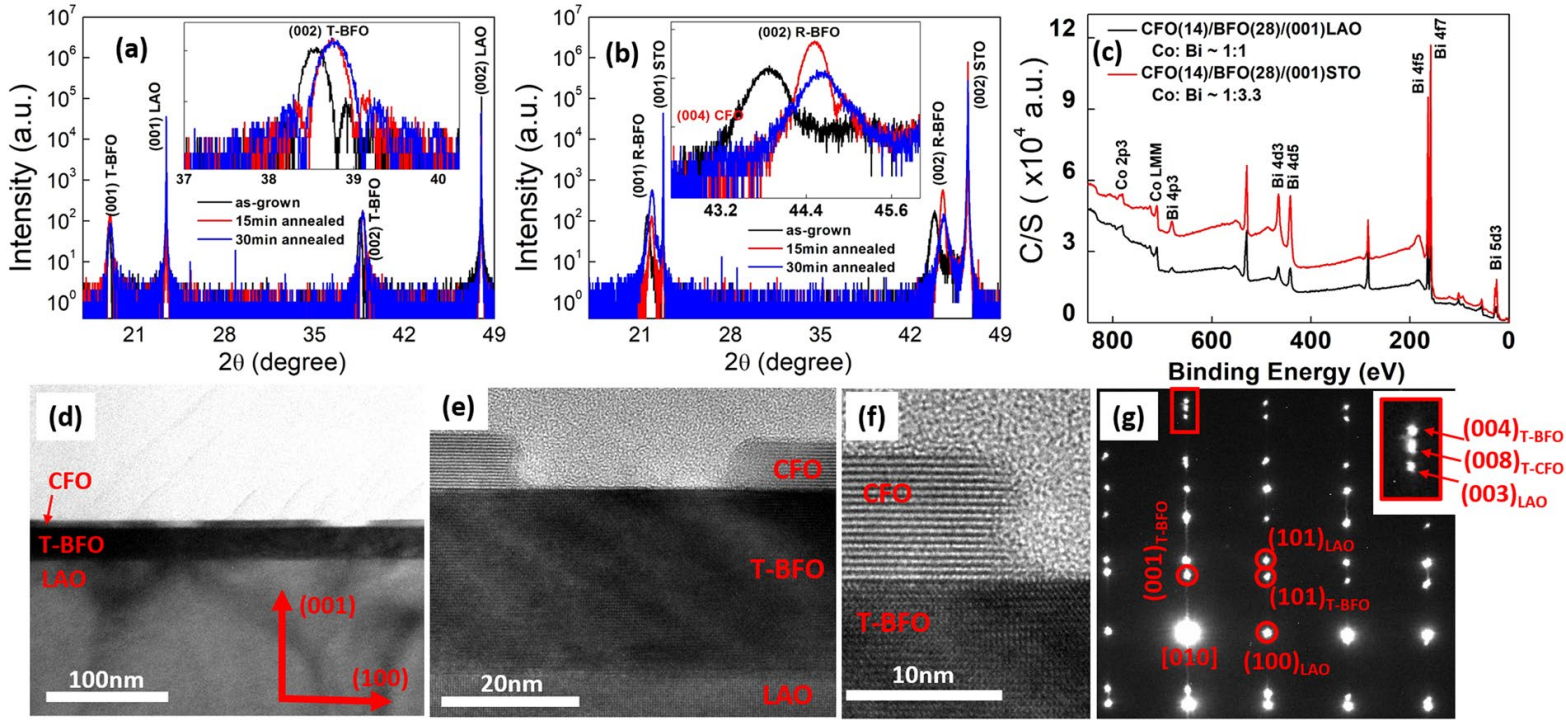
(**a**,**b**) XRD patterns of (**a**) CFO(7 nm)/BFO(28 nm)/(001)LAO, and (**b**) CFO(7 nm)/BFO(28 nm)/(001)STO after different anneal processes. The inset in (**a**) and (**b**) shows the peak shifts caused by post-heat treatments. (**c**) XPS patterns of CFO(14 nm)/BFO(28 nm) nanocomposites. (**d**–**f**) the cross-sectional TEM images of CFO(7 nm)/T-phase BFO nanocomposite under different scales. The crystal orientation is shown in (**d**). (**g**) SAED patterns of (**e**). The zone axis is along [010] direction. The inset in (**g**) is the zoom-in image showing a triplet split along (001) orientation.

Such differences in surface morphologies could be induced by the lattice parameter mismatch between CFO and BFO phases^25^. The X-ray Diffraction (XRD) patterns in Fig. 2(a,b) demonstrate that the BFO deposited on LAO and STO substrates had T-phase and R-phase structures, respectively. In both cases, after annealing, the BFO peaks had a clear shift to higher 2theta values, indicating a significant strain relaxation due to the formation of self-assembled structures. The (004) CFO peaks were only observed on top of the R-phase BFO layer, similar to previous studies of CFO/R-phase BFO nanocomposites^5,13,20^. On top of the T-phase BFO, no such (004) CFO peaks were detectable. Considering that the in-plane crystal lattice mismatch between CFO and T-phase BFO (12%) is much larger than that between CFO and R-phase BFO (5%), it is reasonable to conclude that the CFO on T-phase BFO had a lower crystalline quality^21^. However, the X-ray photoelectron spectroscopy (XPS) patterns in Fig. 2(c) clearly reveal Co peaks, suggesting that CFO may also exist on the top layers^26,27^. Noting that XPS can only detect the top 10 nm of a sample, the fact that the XPS patterns revealed both Co and Bi peaks confirms that the CFO/BFO samples formed some type of self-assembled structure, although CFO and BFO were deposited in separate steps before annealing. The difference in morphologies between CFO/T-phase BFO and CFO/R-phase BFO might be the reason for the different Co/Bi ratios in the XPS analysis given in Fig. 2(c).

Cross-sectional transmission electron microscope (TEM) images given in Fig. 2(d–f) demonstrate that the CFO/T-phase BFO nanocomposites remained as a bilayer structure after annealing. The platform-like topography resulted from a discontinuity in the CFO phase distribution. Figure S2 in the Supplemental materials shows an energy-dispersive X-ray spectroscopy (EDS) line scan reflecting the elemental distribution inside the nanocomposite. In Fig. 2(g), the selected area electron diffraction (SAED) pattern along the [010] zone axis exhibited good epitaxial T-phase BFO patterns on LAO substrate. The lattice parameter of the T-phase BFO along the (001) direction was about 4.65 Å, which is consistent with the XRD results (4.64 Å). Moreover, as shown in the inset of Fig. 2(g), a triplet splitting was found, where one peak belonged to the (008) CFO phase. The calculated (001) interplanar spacing of CFO was about 4.88 Å, which is close to that measured in Fig. 2(f) (4.89Å), but notably larger than that of the cubic CFO’s lattice parameter (1/2a_CFO_ = 4.19 Å). The diffraction pattern of CFO along the (100) orientation was barely distinguishable in these patterns, indicating that the CFO lattice may be under significant in-plane compressive strain that disrupted the periodicity along that direction. Based on these TEM observations, it can be reasonably concluded that the CFO phase on top of the T-phase BFO also has a T-phase-like structure, which may explain why the CFO phase forms a platform-like phase distribution with BFO on LAO substrates, instead of the previously reported nanopillar distribution when deposited on STO.

**Figure 3:**
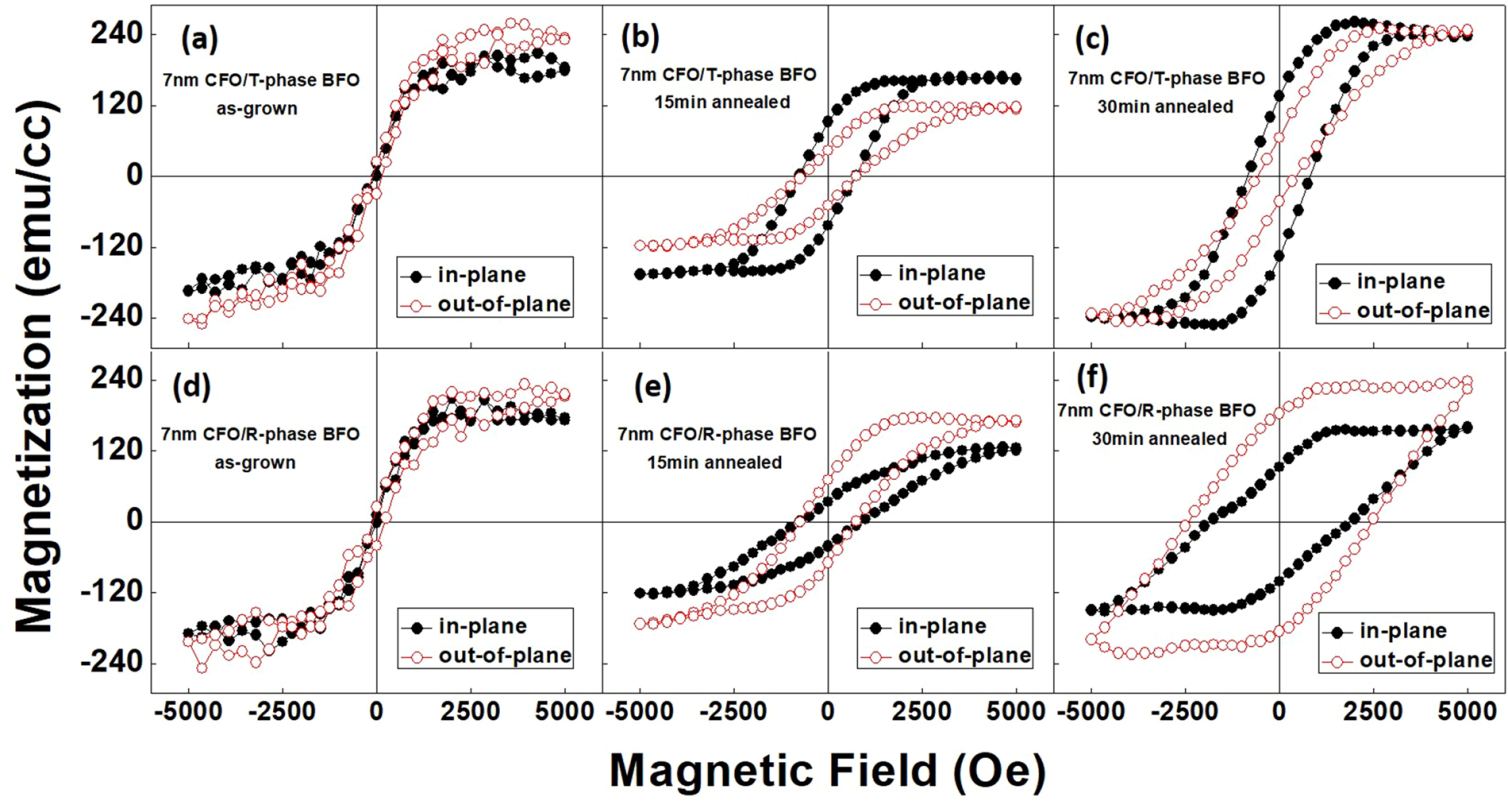
Magnetic hysteresis loops of (**a**–**c**) CFO(7 nm)/BFO(28 nm)/(001)LAO, and (**d**–**f**) CFO(7 nm)/BFO(28 nm)/(001)STO. (**a**,**d**) The as-grown samples, (**b**,**e**) 15 min annealed samples, and (**c**,**f**) 30 min annealed samples.

Figure 3 shows magnetic hysteresis loops measured by a vibrating sample magnetometer (VSM). The magnetization was normalized by the nominal volume of the CFO layer. Since the maximum magnetic field applied was about 5000 Oe, the CFO layers did not reach complete saturation^20^. The as-grown CFO/BFO nanocomposites on either LAO or STO substrates exhibited a similar magnetic isotropy with a small coercivity of less than 120 Oe, indicating without post-heat treatment that the CFO layers have poor crystal quality.

After 15 min annealing, as specific surface morphologies began to develop, the two types of nanocomposites trended towards having opposite magnetic anisotropies. For the CFO/T-phase BFO on LAO, the platform-like topography induced an in-plane (IP) magnetic anisotropy; whereas for the CFO/R-phase BFO on STO, the nanopillar morphology resulted in an out-of-plane (OP) magnetic anisotropy^20,21^. In both cases, the magnetic coercivity was increased to about 800 Oe, indicating an improvement in the CFO phase crystallinity.

When the annealing time was further increased to 30 min, such opposite magnetic anisotropies became more obvious, consistent with trends of the AFM images in Fig. 1, where self-assembled phase distributions became more apparent. In addition, the OP magnetic coercivity of the CFO/R-phase BFO was significantly increased to 2450 Oe, due to a spin-flop coupling at the ferrimagnetic/antiferromagnetic interface^18,20,28^. This coupling effect has been suggested to tilt the antiferromagnetic spins in BFO with respect to the CFO’s spins in the interface region between the two phases during field reversal, enhancing the coercivity and introducing an exchange anisotropy energy. However, under the same annealing condition, the OP magnetic coercivity of the CFO/T-BFO remained low (a more direct comparison of the IP and OP coercivities of the two types of CFO/BFO nanocomposites under different heat treatments is given in Fig. S3 in the Supplementary). This low coercivity was probably caused by the relatively poorer crystallinity of the T-phase-like CFO on T-phase BFO, as evidenced by the XRD and TEM SAED patterns, compared to the cubic-phase CFO with R-phase BFO. However, it may also indicate a change in the magnetization switching mechanism, given that the antiferromagnetic spin state in the T-phase BFO is different from that in the R-phase. The much narrower hysteresis loops of the 7 nm CFO on T-phase BFO demonstrate a much lower energy dissipation on magnetization reversal, which could be beneficial to microelectronic devices.

**Figure 4:**
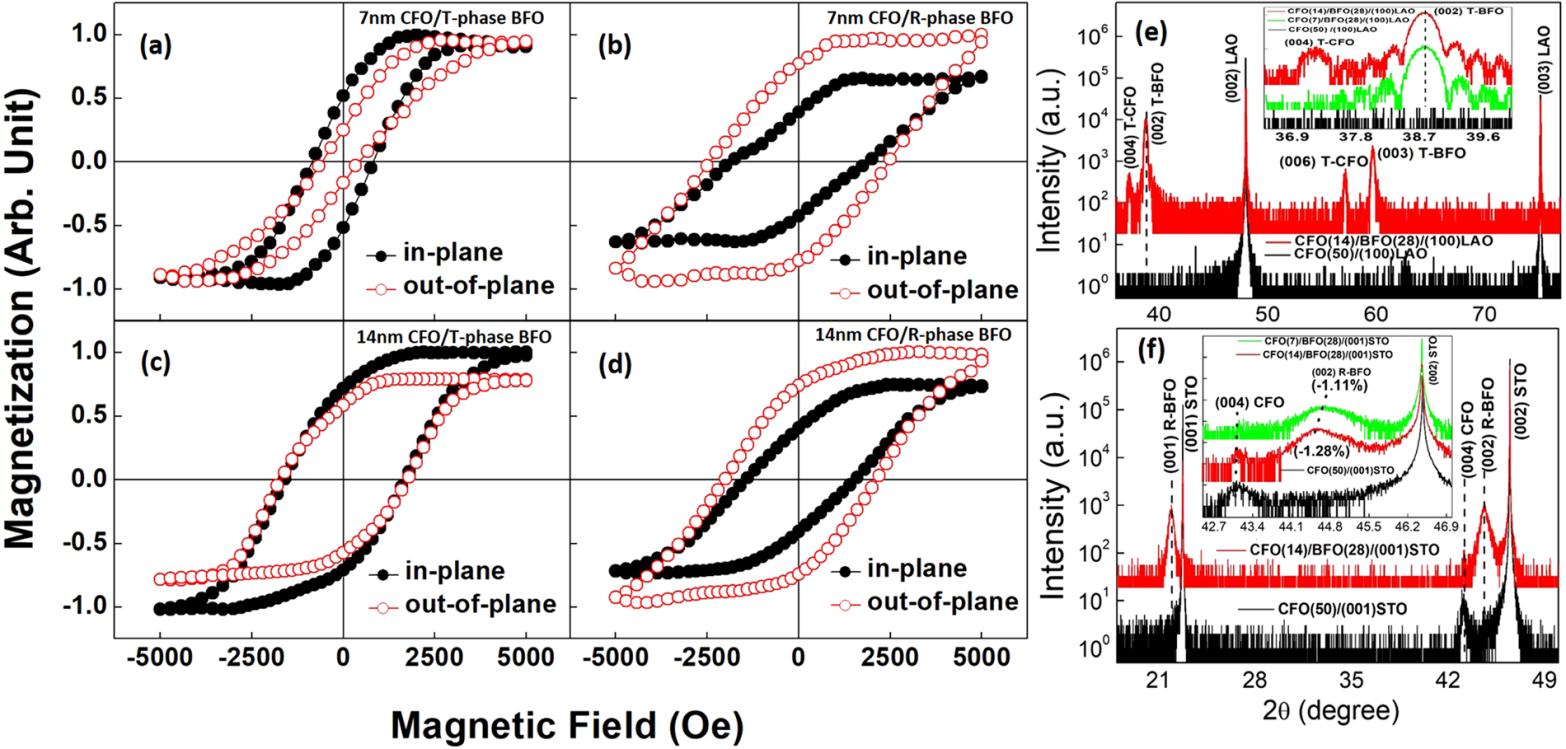
(**a**–**d**) Magnetic hysteresis loops of (**a**,**c**) CFO/T-phase BFO on (001) LAO substrate, and (**b**,**d**) CFO/R-phase BFO on (001) STO substrate. The thickness of CFO was 7 nm in (**a**,**b**), and 14 nm in (**c**,**d**). (**e**,**f**) XRD patterns of (**e**) CFO/T-phase BFO nanocomposites, and (**f**) CFO/R-phase BFO nanocomposites. The insets in (**e**,**f**) show the strain changes in CFO and BFO caused by a thickness factor.

Figure 4(a–c) shows a thickness effect of the CFO layer on the magnetic coercive field, in particular along the OP direction. The CFO/T-phase BFO had a distinctive enhancement in its OP coercivity from 710 Oe to 2120 Oe, as the thickness of the CFO layer increased from 7 nm to 14 nm; whereas, for the CFO/R-phase BFO, its OP coercivity dropped from 2450 Oe to 2090 Oe. At the same time, 14 nm thick CFO on either T-phase or R-phase BFO had similar hysteresis loops, with some anisotropic difference due to the shape anisotropy.

For the CFO/T-phase BFO nanocomposites, the enhancement in the coercivity may be due to its improved crystallinity, as the T-phase CFO peaks can be seen in the XRD patterns of Fig. 4(e) when the thickness of the CFO layer was increased to 14 nm. It is important to point out that without the BFO layer, even a 50 nm CFO layer on (001) LAO did not reveal T-phase peaks. Clearly, in this case, the T-phase BFO plays a key role as the template to induce an epitaxial T-phase CFO on top of it.

On the other hand, for the CFO/R-phase BFO, the decrease of the coercivity can be explained from the perspectives of strain and interface coupling effects. For the strain effect, as the thickness of the CFO is increased, the internal strain of the layer becomes increasingly relaxed. Accordingly, this would decrease the magnetoelastic energy (K_me_), given as:

1 $${{\rm{K}}}_{me}=-\frac{3}{2}\times {\lambda }_{001}\times Y\times {\varepsilon }_{001},$$

where λ_001_, Y, and ε_001_ are the magnetostrictive coefficient, Young’s modulus and OP strain of CFO, respectively^20,29^. The insert in Fig. 4(f) shows that the (004) CFO XRD peak is shifted for the 14 nm layer, indicating reduced strain in the layer. For the interface coupling effect, because the exchange enhancement mentioned above is an interface effect, as the thickness is increased, it is weakened.

**Figure 5:**
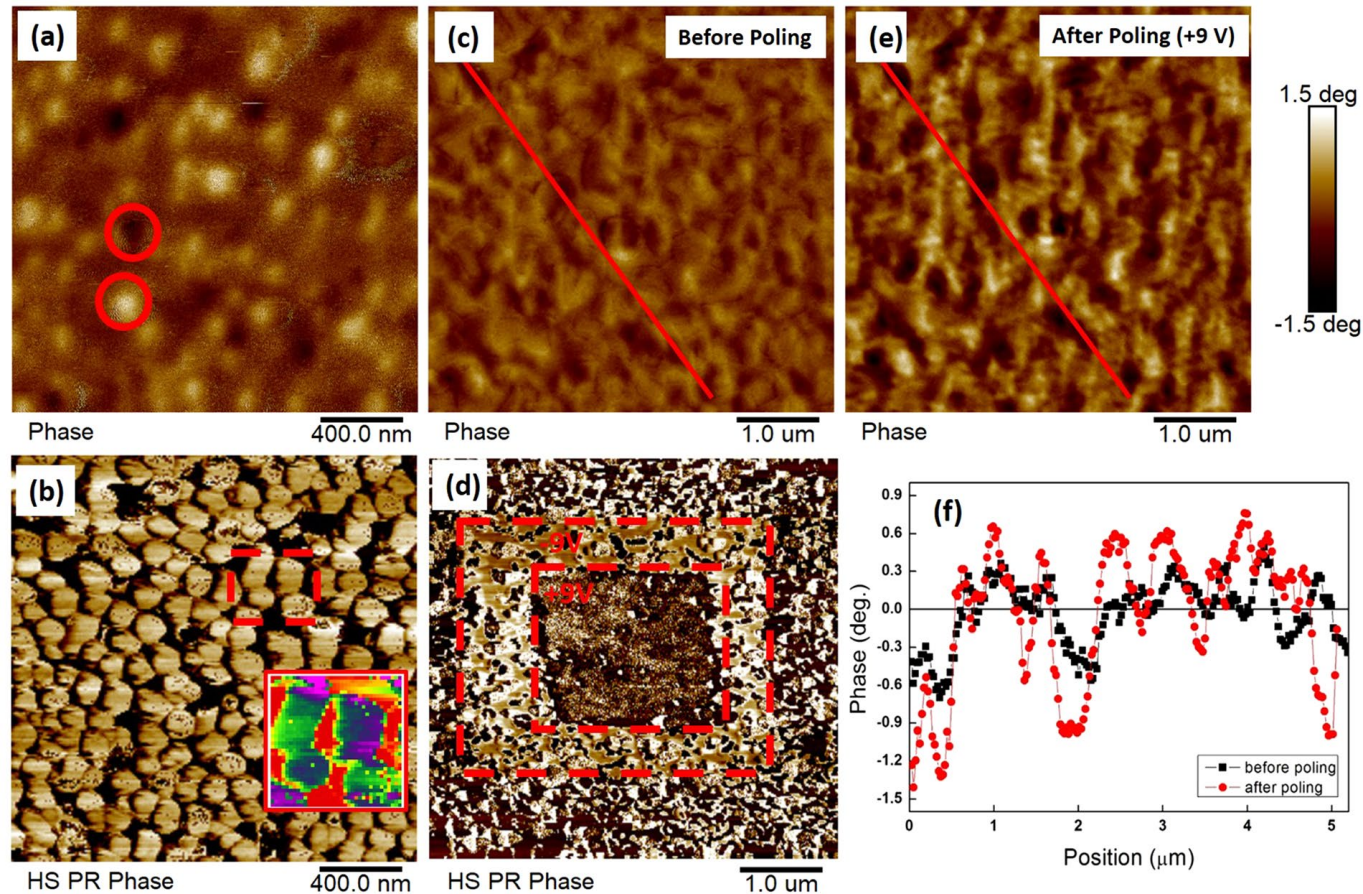
(**a**,**c**,**e**) MFM and (**b**,**d**) PFM phase images of CFO(7 nm)/BFO(28 nm) nanocomposites. (**a**,**b**) CFO/R-phase BFO nanocomposites, and (**c**–**e**) CFO/T-phase BFO nanocomposites. (**f**) Phase profile extract along the red lines in (**c**) and (**e**). The inset in (**b**) is the region in the red box that has a rainbow scale.

Figure 5 presents corresponding magnetic force microscopy (MFM) and piezoresponse force microscopy (PFM) phase images of these two different types of CFO/BFO nanocomposites. The bright and dark contrast in the MFM phase images originated from the stray field of CFO, revealing different magnetic domains with opposite spin orientations. Since the samples were pre-magnetized along the OP direction, and MFM images were recorded at a remanent state, the opposite contrast represents the OP magnetization of CFO. In Fig. 5(a), each nanopillar of the CFO/R-phase BFO sample contained only a single magnetic domain, which is similar to previous reports for CFO-BFO self-assembled nanopillars^30,31^. This is because of shape and size effects, where the nanopillar structure restricts the size of the magnetic domains. However, in Fig. 5(c), each platform of the CFO/T-phase BFO sample can be seen to contain multiple magnetic domains. The platforms were larger in size than the nanopillars, thus a multi-domain structure developed to lower the total energy.

The contrast in the PFM phase images confirms the ferroelectric nature of BFO. In Fig. 5(b), the sphere-like patterns were caused by the protuberant CFO nanopillars rather than the real piezoresponse signal of the R-phase BFO. This may indicate that the BFO matrix was covered by the dense CFO nanopillars^30,32^, similar to the bilayer structure shown in Fig. 2(d). However, the inset in Fig. 5(b) reveals a contrast change (see rainbow scaling), indicative of BFO’s ferroelectricity underneath the CFO nanopillars. Figure 5(d) shows that the CFO/T-phase BFO sample had a mosaic domain pattern. Although CFO platforms covered part of the surface, small regions of bright and dark contrast represented opposite [001] polarizations of the T-phase BFO. The box-in-box pattern at the center illustrates the tunability of the ferroelectric polarizations inside the CFO/T-phase BFO nanocomposite.

Finally, in Fig. 5(c,e), MFM scans are shown before and after poling by a +9 V tip bias. The MFM phase profile in Fig. 5(f) shows that the magnetic phase signal was tuned by the voltage. This increased phase contrast with some domain pattern changes can be attributed to the ME coupling between CFO and T-phase BFO at the interfaces^33^. The more highly aligned ferroelectric polarization in the T-phase BFO may result in better spin alignment in the CFO phase via strain, leading to the enhancement of the magnetization^34^. An exchange coupling should also be considered, where the reorientation of ferroelectric polarization affects the antiferromagnetic spin distribution inside the BFO phase, which could influence the femimagnetic spin arrangement in the CFO^11,24,35^. Similar enhancements were observed when applying a −9V tip bias, for the [001] and $[00\bar{1}]$ ferroelectric polarizations of the T-phase BFO are actually equivalent.

In summary, epitaxial T-phase CFO/BFO nanocomposites were deposited on (001) LAO substrates by PLD. The nanocomposites developed a platform-like topography, which was different from the CFO/R-phase BFO nanopillar structure previously reported when deposited on (001) STO. A strong thickness effect of the CFO layer on the coercive field was found, where a 7 nm CFO layer on the T-phase BFO exhibited a narrow M-H hysteresis loop. The platform T-phase CFO contained multiple magnetic domains whose contrast could be tuned by applying a tip bias. We explain these observations based upon a combination of shape, strain, and exchange coupling effects.

## Methods

The CFO and BFO layers were deposited as individual layers on both single-crystal (001) LAO and (001) STO substrates. The CFO thickness was varied from 7 nm to 14 nm, whereas the BFO thickness was set at 28 nm. In order to prevent Bi evaporation, the deposition temperature was kept at 923 K. The oxygen partial pressure was fixed at 90 mtorr for the deposition of BFO, and at 100 mtorr for CFO. Before deposition of CFO, the as-grown BFO films were *in-situ* annealed in a pure O_2_ atmosphere of 100 mtorr at 923 K for 30 min. To investigate the annealing effect on the CFO layer, after the deposition of CFO, the as-grown films were *in-situ* annealed in a pure O_2_ atmosphere of 100 torr at 998 K for 0 min, 15 min, or 30 min. Crystal structures were determined using a Philips X’pert high resolution X-ray diffractometer (XRD) equipped with a two bounce hybrid monochromator, and an open three-circle Eulerian cradle. The surface topologies were observed by a Veeco SPI 3100 atomic force microscope (AFM). Piezoresponse force (PFM) and magnetic force (MFM) modes were used to image the ferroelectric and magnetic domains, respectively, as well as to detect the change of magnetic signals under a tip bias. To realize the PFM measurements, a 5 nm SrRuO_3_ (SRO) buffer layer was deposited between the BFO layer and substrate by PLD. The magnetic hysteresis loops were measured with a Lakeshore 7300 series vibrating sample magnetometer (VSM) system. The surface composition was semiquantitatively analyzed using X-ray photoelectron spectroscopy (XPS, PHI Quantera SXM). A JEOL 2100 transmission electron microscope (TEM) was employed to observe the cross-sectional images of the CFO/T-phase BFO nanocomposites. The energy-dispersive X-ray spectroscopy (EDS) line scans were taken by a silicon drift detector-based EDS system integrated in the TEM.

### Data availability statement

All data generated or analysed during this study are available from the corresponding author on reasonable request.

## Electronic supplementary material

Supplementary Material
