# Supplementary material for: Magnetoelectricity of CoFe2O4 and tetragonal phase BiFeO3 nanocomposites prepared by pulsed laser deposition
Source: Sci Rep. 2018 Jan 10;8:323. doi: 10.1038/s41598-017-18788-8 (PMC5762771; doi:10.1038/s41598-017-18788-8)
Supplement: Supplementary file 1 — Supplementary Material [file 41598_2017_18788_MOESM1_ESM.pdf]

Magnetoelectricity of $CoFe_2O_4$ and tetragonal phase $BiFeO_3$ nanocomposites prepared by pulsed laser deposition

Min Gao*, Ravindranath Viswan, Xiao Tang, Chung Ming Leung, Jiefang Li, and D. Viehland

Department of Materials Science and Engineering, Virginia Tech, Blacksburg, VA 24061, U.S.A

*Corresponding author (email: gaom9016@vt.edu)

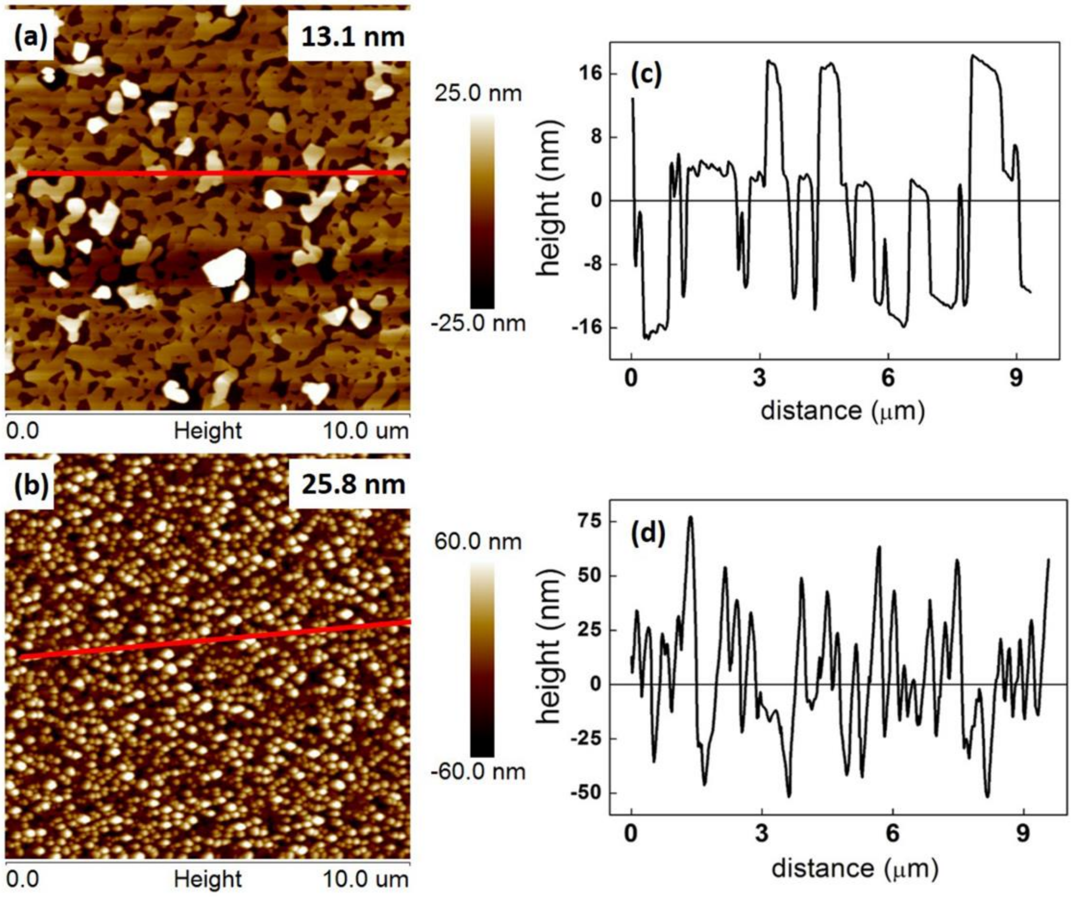

Figure S1. AFM height images of (a) CFO(7nm)/T-phase BFO nanocomposites, and (b) CFO(7nm)/R-phase BFO nanocomposites. The surface roughnesses are given in the insets. (c) and (d) are the profile images extracte along the red lines of (a) and (b), respectively.

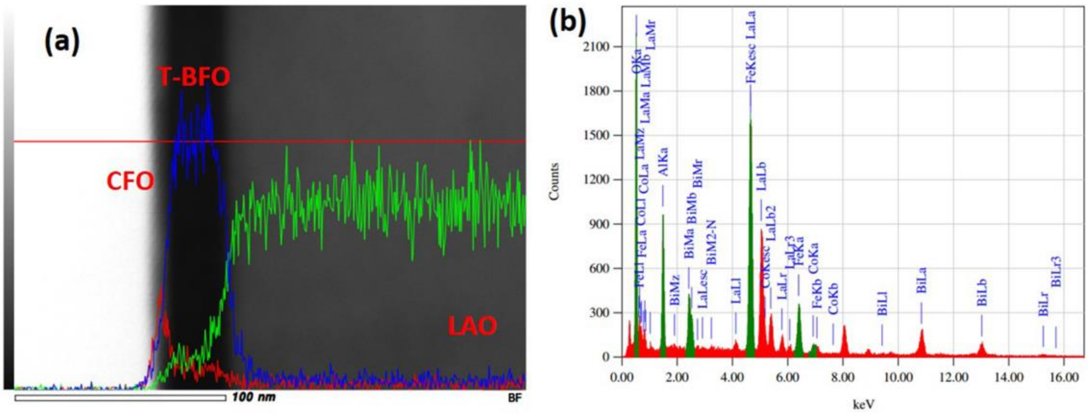

Figure S2. Energy-dispersive X-ray spectroscopy (EDS) line scan of CFO(7nm)/T-phase BFO nanocomposite. The red, blue, and green lines in (a) represented Co, Bi, and Al elements, respectively.

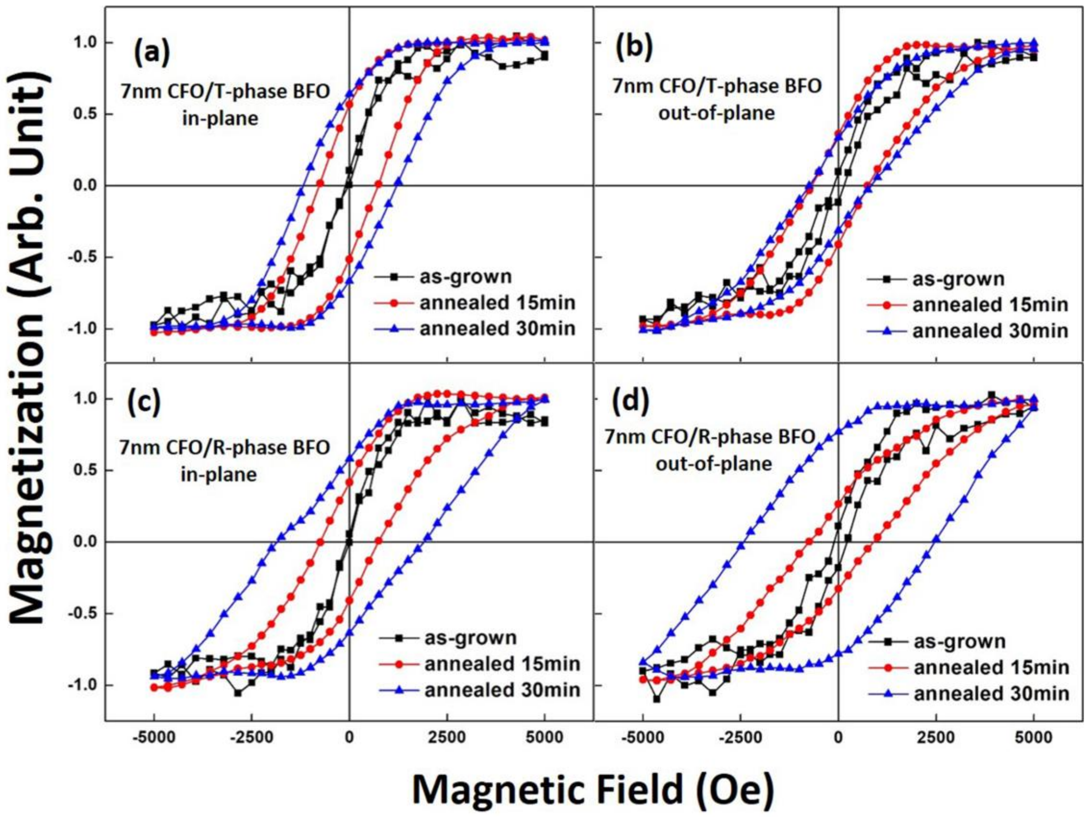

Figure S3. **Magnetic hysteresis loops of (a), (b) CFO(7nm)/T-phase BFO(28nm) nanocomposites, and (c), (d) CFO(7nm)/R-phase BFO(28nm) nanocomposites after different annealing conditions. (a), (c) Measurements along in-plane direction, and (b), (d) measurements along out-of-plane direction.**
